# Clinical characteristics and genetic spectrum of 26 individuals of Chinese origin with primary ciliary dyskinesia

**DOI:** 10.1186/s13023-021-01840-2

**Published:** 2021-07-01

**Authors:** Xinyue Zhao, Chun Bian, Keqiang Liu, Wenshuai Xu, Yaping Liu, Xinlun Tian, Jing Bai, Kai-Feng Xu, Xue Zhang

**Affiliations:** 1 McKusick-Zhang Center for Genetic Medicine, State Key Laboratory of Medical Molecular Biology, Institute of Basic Medical Sciences, Chinese Academy of Medical Sciences and Peking Union Medical College, Beijing, 100005 China; 2 Department of Pulmonary and Critical Care Medicine, State Key Laboratory of Complex Severe and Rare Diseases, Peking Union Medical College Hospital, Chinese Academy of Medical Sciences and Peking Union Medical College, Beijing, 100730 China; 3 Department of Pulmonary and Critical Care Medicine, The First Affiliated Hospital of Guangxi Medical University, Nanning, China

**Keywords:** Primary ciliary dyskinesia, Clinical characteristics, Genetic spectrum, Chinese origin

## Abstract

**Background:**

Primary ciliary dyskinesia (PCD) is a rare, highly heterogeneous genetic disorder involving the impairment of motile cilia. With no single gold standard for PCD diagnosis and complicated multiorgan dysfunction, the diagnosis of PCD can be difficult in clinical settings. Some methods for diagnosis, such as nasal nitric oxide measurement and digital high-speed video microscopy with ciliary beat pattern analysis, can be expensive or unavailable. To confirm PCD diagnosis, we used a strategy combining assessment of typical symptoms with whole-exome sequencing (WES) and/or low-pass whole-genome sequencing (WGS) as an unbiased detection tool to identify known pathogenic mutations, novel variations, and copy number variations.

**Results:**

A total of 26 individuals of Chinese origin with a confirmed PCD diagnosis aged 13 to 61 years (median age, 24.5 years) were included. Biallelic pathogenic mutations were identified in 19 of the 26 patients, including 8 recorded HGMD mutations and 24 novel mutations. The detection rate reached 73.1%. *DNAH5* was the most frequently mutated gene, and c.8383C > T was the most common mutated variant, but it is relatively rare in PCD patients from other ethnic groups.

**Conclusion:**

This study demonstrates the practical clinical utility of combining WES and low-pass WGS as a no-bias detecting tool in adult patients with PCD, showing a clinical characteristics and genetic spectrum of Chinese PCD patients.

**Supplementary Information:**

The online version contains supplementary material available at 10.1186/s13023-021-01840-2.

## Introduction

Motile cilia and flagella are highly conserved organelles that have a well-organized ‘9 + 2’ axoneme—9 peripheral microtubule doublets and a central pair of microtubule doublets—associated with multiprotein ultrastructure components, such as outer dynein arms, inner dynein arms, nexin-dynein regulatory complexes, and radial spokes rhythmically bending and beating in a proper pattern to expel the flow of extracellular fluid [1, 2]. Primary ciliary dyskinesia (PCD, OMIM ID: 244400), which is characterized by the impaired function of motile cilia, is a rare, genetically heterogeneous, autosomal recessive or X-linked disorder with an estimated incidence of 1:10,000–1:20,000 [3]. PCD has a wide range of clinical manifestations, including neonatal respiratory distress, year-round cough productive of sputum, persistent otitis media, bronchiectasis, infertility, and left–right laterality defects [4]. A specific subset of PCD, also known as Kartagener Syndrome (KS), is characterized by bronchiectasis, nasosinusitis and situs inversus [2].

As our understanding of genetics and clinical diagnosis of PCD far improves, multiple pathogenic genes of PCD have been reported, most of which encode axonemal, cytoplasmic, and regulatory proteins involved in the assembly, structure, and function of motile cilia, and the number of causative genes identified to date has significantly increased to 51 [5]. Specific PCD pathogenic genes cause distinct clinical symptoms and disease severities, making PCD a highly heterogenous disease and contributing to challenges for definitive diagnosis [4]. Some pathogenic genes lead to specific ultrastructural defects in the ciliary axonemal structure, which can be diagnosed by transmission electron microscopy (TEM) [1, 4, 6]. Therefore, TEM was a gold standard diagnostic test for PCD until the finding that some genes, such as *DNAH11*, *CCDC65*, and *DRC1*, give rise to PCD symptoms with normal cross-sections of the motile cilia axoneme [7–9]. It is predicted that motile ciliary cross section defects can be detected in 70% of PCD cases [6, 10]. It is hard to diagnose PCD for there is no definitive definition of PCD and no strictly agreed criteria for PCD testing. The diagnosis of PCD may require combinations of copious tests, including nasal nitric oxide (nNO) measurement, digital high-speed video microscopy with ciliary beat pattern analysis (HSVA), immunofluorescence (IF) imaging for specific axonemal proteins, and gene testing [4, 11, 12]. Internationally, nNO measurement is 98% sensitive and 99% specific (cut-off value is 77 nL/min) for identifying individuals with PCD [2]. However, nNO measurement has only been available in China in the past 5 years, and the only related research showed that the best sensitivity is 86.1% and specificity is 91.4% (cut-off value is 76 nL/min) [13]. Given the lack of an agreed standard for nNO measurement or optimal cut-off values in a Chinese population, nNO measurement may suggest the presence of PCD but cannot be considered diagnostic in China. PICADAR, which is a score, contains characteristics of daily wet cough that started in early childhood, chest symptoms in the neonatal period, neonatal unit experience, situs abnormality, congenital heart defect, persistent perennial rhinitis, and chronic ear or hearing symptoms; and represents a simple diagnostic clinical prediction rule with good accuracy and validity for PCD [14]. Nevertheless, some of our patients were either older at presentation, or were born at home without significant medical contact in the neonatal period, meaning the use of PICADAR would not have been applicable. HSVA had excellent 96% sensitivity and 91% specificity to diagnose PCD [15]. However, methods for HSVA including sampling techniques, microscopes, cameras, temperature during analysis, software and evaluation criteria differ among centers [3]. A lack of standardized protocol restricts the application of HSVA in PCD diagnosis in isolation. In previous study, Immunofluorescence successfully identified 22 of 25 patients with PCD and normal staining in all 252 who were highly unlikely to be considered PCD, showing a high specificity but a limited sensitivity [16]. There is no single gold standard for PCD diagnosis so far [4, 10].

As the number of pathogenic genes underlying PCD rapidly increases to over 50 to date, the use of a genetic panel is hindered due to disadvantages, such as the limited gene-detection number, slow gene-updating speed, high renewing price, and unavailability to unknown candidate genes. A recent study demonstrated only a 67.6% detection rate for PCD when using a genetic panel targeted for 26 known PCD relevant genes and 284 additional candidate genes [17]. Compared with genetic panel, WES is more expensive but is associated with in an improved diagnostic rate (1.25 times of genetic panel's diagnostic yield) [18]. Meanwhile, the cost of WES and WGS has been decreased rapidly [18, 19]. In our cohort, the cost of WES and low-pass WGS of one patient is 1500RMB and 2100RMB, respectively. Hence, WES combined with low-pass WGS is our best choice to rapidly identify pathogenic/likely pathogenic single nucleotide variations (SNVs) and copy number variations (CNVs) in expanded candidate genes.

In this study, we demonstrated the practical PCD diagnostic criteria, emphasized the use of WES and low-pass WGS in combination to detect putative deleterious variations in 51 known PCD-causing genes and 42 suspected pathogenic genes, and supported the cost-saving potential of diagnostic low-pass WGS.

## Results

### Demographic data of patients with PCD at Peking Union Medical College Hospital (PUMCH)

**Fig. 1:**
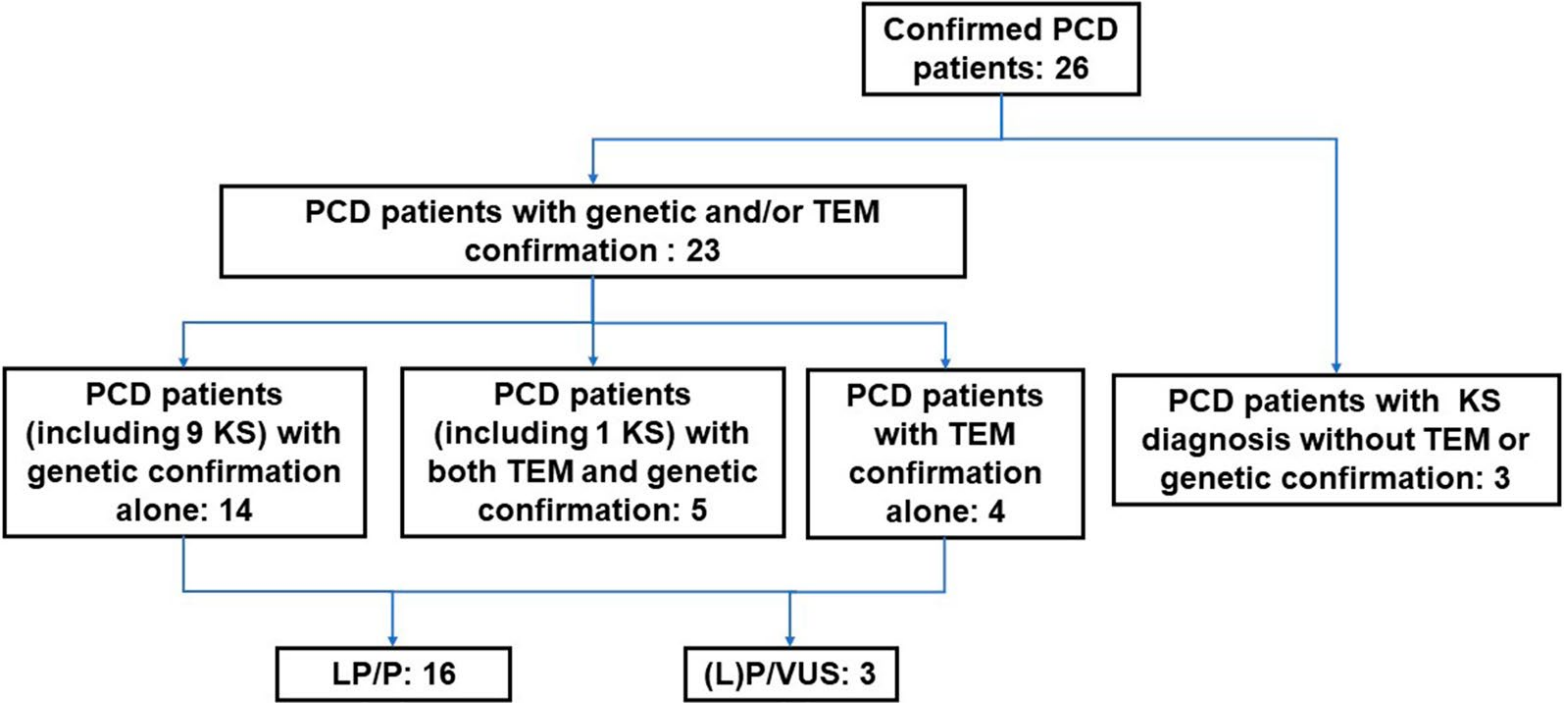
Diagram outlining the included patients. KS, Kartagener syndrome; TEM, Transmission electron microscopy; P, pathogenic; LP, likely pathogenic; VUS, variants uncertain significance

We performed a retrospective study of patients with a high-suspicion of PCD who had attended PUMCH between 2012 and 2020 (Fig. 1). 26 patients were included, with an age at diagnosis which ranged from 13 to 61 years-old (median age, 24.5 years) (As shown in Additional file 2: Table S1). Among the 26 patients, 16 had an age at symptom onset ranging from newborn to 23 years (median age, 1.5 years), and the age of the remaining 10 patients was not available. Male and female patients comprised 46.2% and 53.8% of the sample, respectively. Two patients, who were brother and sister, had a family history of PCD, and six patients (23.1%) had a suspected family history, some of whose family members had a history of asthma or chronic bronchitis. None of their parents were consanguineous.

### Main characteristics of the 26 patients with confirmed PCD

Patients were diagnosed with PCD based on KS diagnosis (13/26, 50%), biallelic mutations in one of the known causative genes (19/26, 73.1%) or ciliary ultrastructural defects shown on TEM (7/26, 26.9%). Among the patients, 10 (38.5%) had both KS and biallelic causative gene mutations, and 4 (15.4%) patients had both ciliary ultrastructural defects and biallelic causative mutations. More details for the clinical characteristics and pathogenic genes of the 26 patients with confirmed PCD are shown in supplementary files.

### Clinical data of the patients

A total of 2 patients had no data on respiratory symptoms, and in the other 24 patients (except 1 with incomplete data), symptoms of recurrent fever (11/24, 45.8%), cough (24/24, 100%), productive cough (23/24, 95.8%), hemoptysis (7/24, 29.2%), dyspnea after exercise (7/23, 30.4%) and dyspnea at rest (1/23, 4.3%) occurred. Of the 23 patients with complete data, 3 patients (13.0%) had a history of otitis media, and 19 of the 24 patients (79.2%) had a history of nasosinusitis. One female patient had been formally diagnosed as infertility (previously diagnosed as polycystic ovary syndrome), and another female patient experienced a spontaneous abortion. Among the 12 male patients, 7 of them underwent sperm testing, among whom 6 patients had abnormal results (85.7%).

Of the 18 patients (44.4%) who underwent pulmonary function tests, 8 had a result of forced expiratory volume in one second/forced vital capacity (FEV_1_/FVC) < 70%, which suggests that they had obstructive ventilatory dysfunction. In 5 patients who had data for total lung capacity% (TLC%) and diffusing capacity of the lungs for carbon monoxide% (DLCO%), 1 patient’s TLC% was less than 80%, showing restrictive ventilatory dysfunction, and 1 patient’s DLCO% was less than 80%, showing impaired diffusion capacity. One patient had both restrictive ventilatory and obstructive ventilatory dysfunction.

The results of microbial cultures of respiratory secretions were collected in 19 patients. Nine patients were negative (9/19, 47.4%), while the other ten were positive (10/19, 52.6%). Among the positive results, *Pseudomonas aeruginosa* was the most common organism (7/19, 36.8%). There were also patients with *Haemophilus influenzae*, *Staphylococcus aureus*, and *Haemophilus influenzae* combined with *Streptococcus pneumoniae* (1/19, 5.3%).

**Fig. 2:**
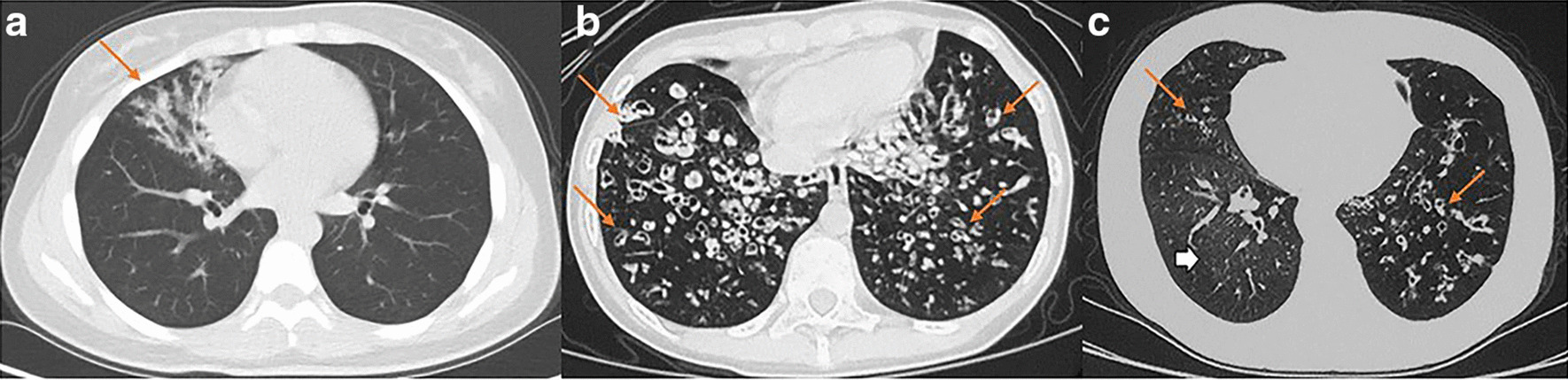
The bronchiectasis of patients by CT scan.** a** Bronchiectasis in the right middle lobe in patient 11.** b** Diffuse bronchiectasis in the right middle lobe, lingular segment, and both lower lobes in patient 14.** c** Bronchiectasis in the right middle lobe and left lower lobe (red arrows) in patient 20. In addition, air trapping was observed in his right lower lobe (white arrowhead)

A total of 24 patients underwent computed tomography (CT) evaluation (Fig. 2). One was normal, 18/24 (75%) demonstrated bronchiectasis in one or more lobes, and 5/24 (20.8%) showed all lobes affected. Incidence of bronchiectasis in the right middle lobe, right lower lobe, left lingular segment and left lower lobe was similar (83.3%, 87.5%, 83.3%, and 83.3% respectively), while the right upper lobe and anterior segment of left upper lobe were less commonly affected (29.2%, 33.3% respectively).

**Fig. 3:**
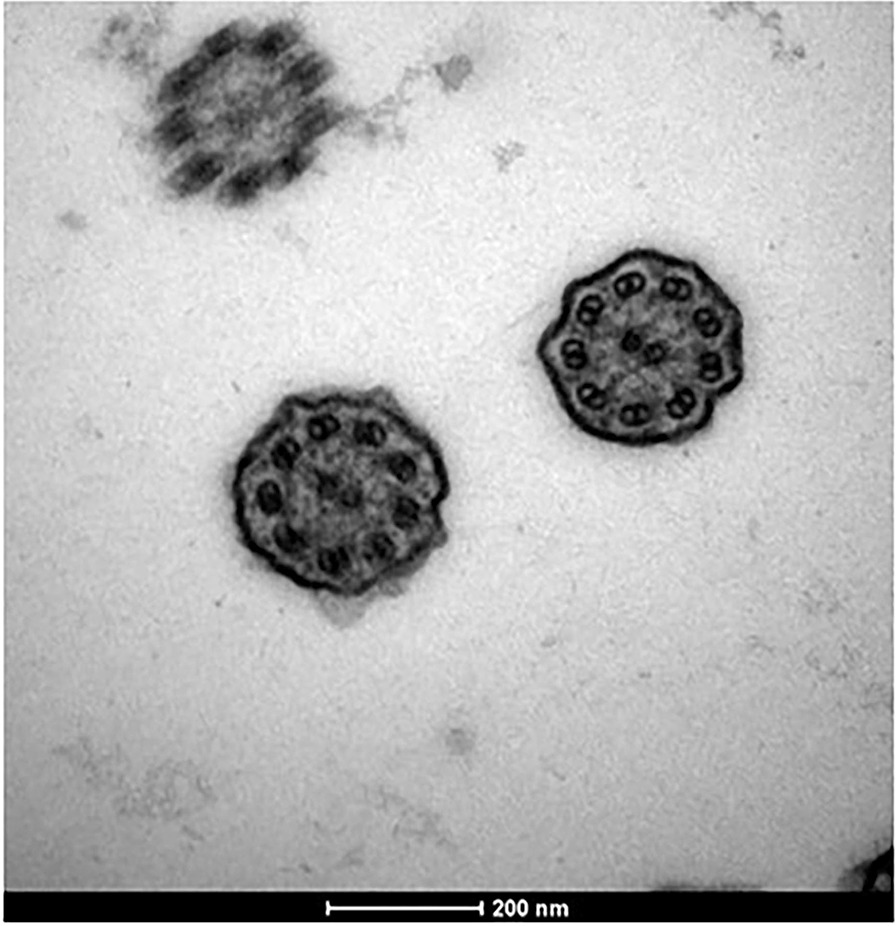
TEM result of patient 8. The TEM result shows wide inner dynein arm defects and shortness or defect of the outer dynein arms in patient 8

Nine patients had TEM data, of whom seven patients (77.8%) had ciliary ultrastructural defects. Three patients (3/9, 33.3%) showed an absence of cilia. Three patients (3/9, 33.3%) showed inner dynein arms defects (and intact outer dynein arms). Patient 8 (1/9, 11.1%) showed both inner and outer dynein arms defects (Fig. 3).

### Detection rate and characteristics of pathogenic mutations

**Fig. 4:**
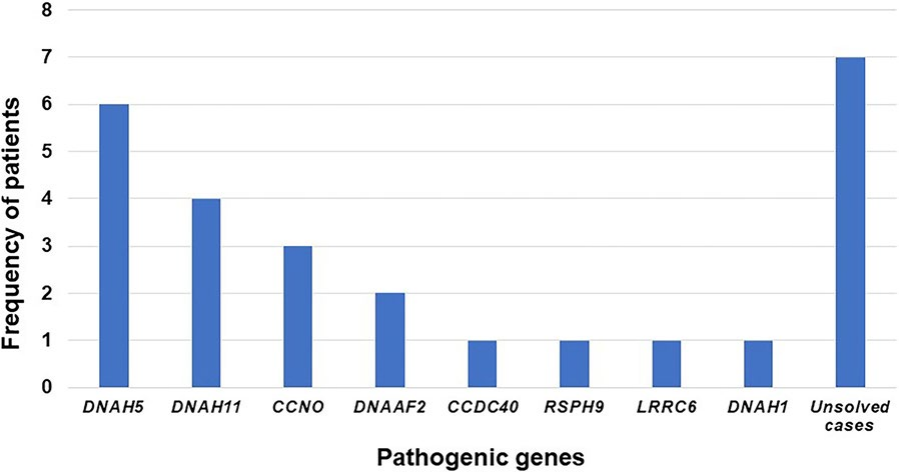
Characteristics of detected pathogenic genes in patients of Chinese origin with PCD.* DNAH5* was the most prevalent. Two patients with mutated* DNNAF2* were siblings

A total of 26 patients with PCD underwent WES in combination with low-pass WGS as described in the methods. Consistent with recessive inheritance, 19 individuals with PCD carried biallelic pathogenic mutations in at least 1 of 93 candidate genes (51 known plus 42 suspected), revealing a relatively high genetic detection rate of 73.1%. A total of 32 different variants in 8 known pathogenic genes were identified, with 8 known HGMD disease-causing and 24 novel mutations (genetic details are shown in Additional file 2: Tables S1). No biallelic pathogenic mutations in suspected PCD genes were found in our patients. Among the detected genes, *DNAH5*, encoding the dynein arms of cilia, was carried by 6 (6/26, 23.1%) patients with PCD, making it the most prevalent pathogenic gene (Fig. 4) [20]. The second most prevalent pathogenic gene was *DNAH11*, which also encodes the ciliary outer dynein arm protein [21]. Loss-of-function mutations in *DNAH11* affected 4 unrelated patients, accounting for 15.4% of all patients with confirmed PCD. An interesting finding in our study was that the c.8383C > T (p.R2795X) mutation in *DNAH5* occurred at rather high frequency in all 26 patients with definitive PCD. Three unrelated patients carried this mutation, including two patients (patient 6 and 14) with compound heterozygous mutations and one patient (patient 4) with homozygous mutations without any known consanguineous marriages.

### Pathogenic/benign variant analysis

**Fig. 5:**
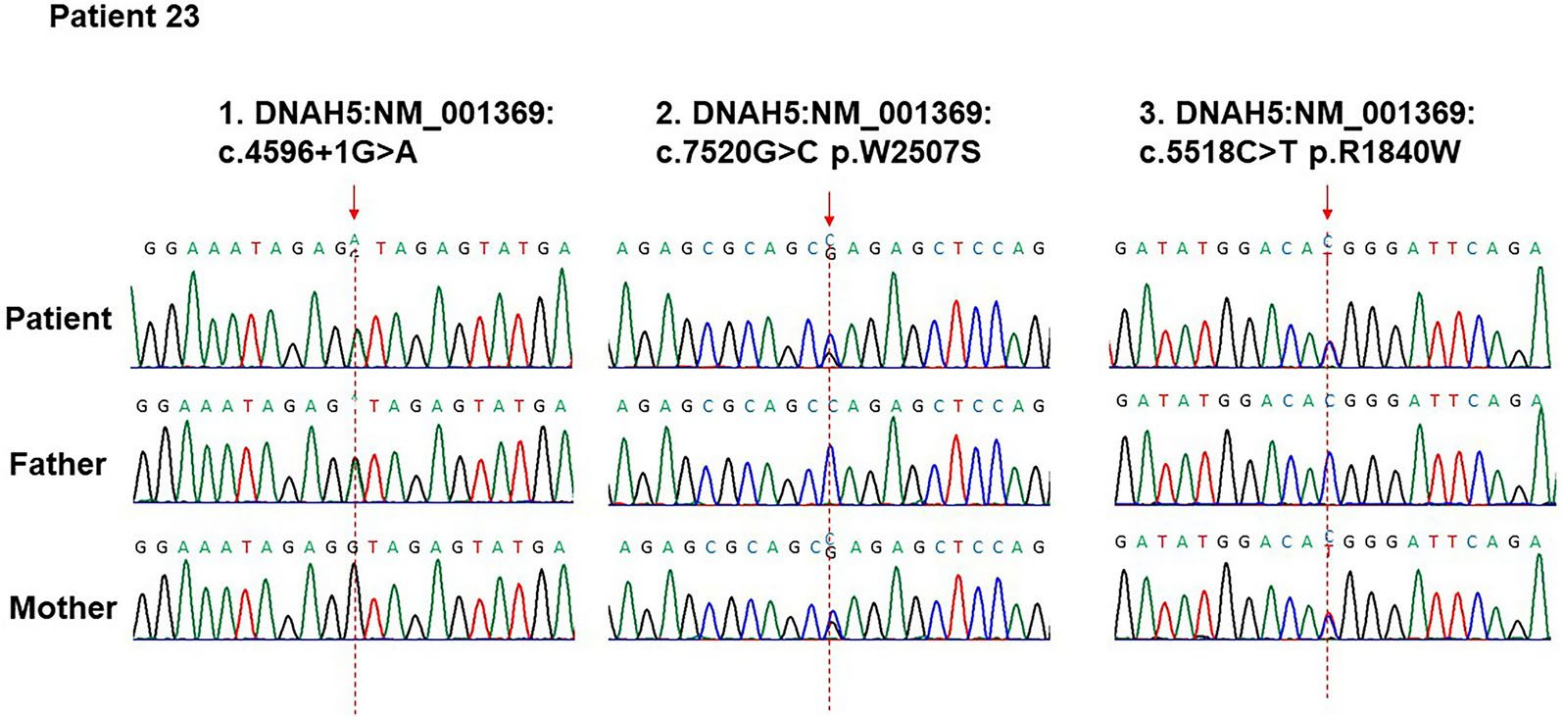
Sanger sequencing and parental original confirmation of mutations in patient 23

Of the 24 novel mutations found in our patients, 19 were loss-of-function mutations, including stop-loss, stop-gain, frameshift, and splice site defects, and consequently caused functional defects of the gene products. One c.7769G > A (p.G2590D) missense variant in *DNAH5* that was not observed in the ExAC, GnomAD, 1000 genomes and ESP6500 databases was predicted to be likely pathogenic by ACMG-AMP standards (InterVar, http://wintervar.wglab.org/). These aforementioned 20 variations were regarded to be putative deleterious and disease-causing in our study. Apart from the abovementioned data, the remaining 4 variants of uncertain significance (VUS) assessed by ACMG were equivocal. Therefore, VarCards was chosen for further analysis, offering a damaging score evaluated by the portion of pathogenic predictions provided by 23 algorithms. The c.9631C > T (p.P3211S) VUS in *DNAH11* carried by patient 25 was considered to be deleterious by VarCards with a damaging score of 0.91. For patient 13, the c.53T > C (p.I18T) VUS in *LRRC6* had a score of 0.83. In patient 23, two VUS, c.7520G > C (p.W2507S) and c.5518C > T (p.R1840W) in the same allele of *DNAH5* (Fig. 5) had damaging scores of 0.57 and 0.61, respectively. These 4 VUS were deemed to be harmful by VarCards.

### CNV detection in a patient with PCD by WES and low-pass WGS

**Fig. 6:**
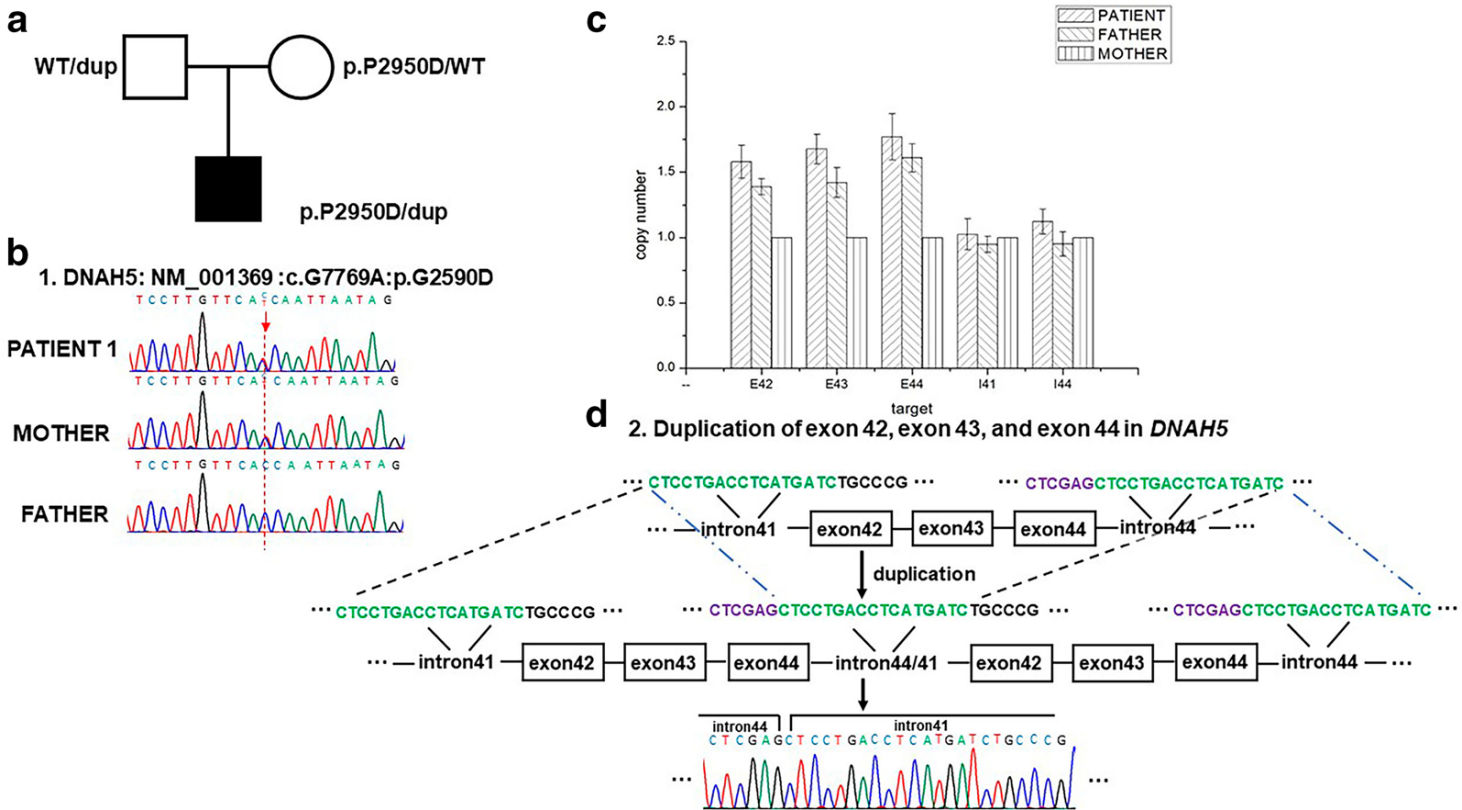
The CNV identified in patient 1.** a** The family pedigree chart for patient 1.** b** The Sanger sequencing results of c.G7769A in DNAH5. The mutation was of maternal origin.** c** The gDNA qPCR result, showing duplication of exon 42, exon 43, and exon 44 of DNAH5 in the patient and his father.** d** Schematic diagram and Sanger sequencing confirmation of the duplication CNV

Patient 1 was detected to carry c.7769G > A (p.G2950D) in *DNAH5* by WES, which was assumed to be pathogenic by ACMG/AMP. Sanger sequencing of both parents showed the mutation was of maternal origin. As WES was not sensitive to a large insertion or deletion of DNA fragments, 10xWGS was performed in this patient to detect the second hit, presumably a CNV, in *DNAH5*. With the WGS results, a duplication of *DNAH5* encompassing exon 42, exon 43, and exon 44 was revealed. This CNV was verified to be of paternal origin by means of gDNA qPCR (Fig. 6). The pathogenicity of this duplication was explicit, as exon 42, exon 43, and exon 44 of *DNAH5* included 566 nucleotides and caused a frameshift of ORF.

## Discussion

During recent years, more and more genes relevant to PCD have been identified due to our further comprehension of PCD and decreasing price for next generation sequencing. The number of known PCD-causative genes has grown to 51, and this number is still growing. PCD genetic panels with a limited number of genes tested are no longer practical for PCD diagnosis, and other recently developed methods to diagnose PCD, such as nNO, HSVA and three-dimensional structured illumination microscopy or stochastic optical reconstruction microscopy for PCD protein dysfunction assessment, can be expensive, unavailable, or difficult to standardize [11, 12]. Here, we used a process combining clinical symptoms and WES accompanied by low-pass WGS, which provides a unbiased portrayal of potentially causative variants and helps reveal CNVs that are not sensitive to WES, to help confirm PCD diagnosis. This diagnostic approach was an alternative and convenient diagnostic way for PCD patients who were reluctant to undergo invasive procedures such as bronchoscopy to obtain tissue for HSVA, IF, TEM. In our study, the mutation detection rate was as high as 73.1%, which slightly exceeds the previous 70% genetic detection rate [6]. This practical molecular genetic technique was also demonstrated to reveal the mutation characteristics of 76% of patients with PCD in a study by Marshall [22]. WES in combination with low-pass WGS has become a vital auxiliary tool for clinicians regarding disease diagnosis, decision-making and genetic precaution. For some patients with uncertain diagnoses, genetic screening can enable earlier recognition of PCD and consequently, appropriate treatment which will benefit patients and improve their quality of life. Even for those patients with typical PCD phenotypes such as KS, application of WES/WGS is still necessary as an auxiliary confirmation, for KS alone has not been classified as a standard criterion of PCD confirmation [4]. What’s more, WES/WGS can help to identify novel mutations, facilitate diagnosis of additional PCD cases with the same variants, help future prenatal genetic consulting and reveal hotspots for future gene therapy. New gene therapy approaches have been applied using lentiviral transduction and transcription activator-like effector nucleases (TALEN) technology for restoration of gene expression in vitro during recent years [23]. High-frequency mutation c.8383C > T (p.R2795X) in *DNAH5* could be a hotspot and candidate target for gene therapy in Chinese PCD patients. However, we should also keep in mind that genetic screening cannot fully supplant specialized center diagnosis so far. Genetic testing has its limitation in poorly described gene variants, and genetic causes of about one-third PCD patients still remain unsolved [1].

PCD is a highly heterogeneous disease whose diagnosis can be obscure, and the age at onset is difficult to ascertain partly because of its atypical clinical manifestations and nonawareness of the disorder for most of the physicians in basic hospitals in China. In this study, the median age at diagnosis of 24.5 years was relatively high in all patients with suspected PCD, which may have mostly been due to selection bias because PUMCH is a hospital for adults. Selection bias also explains the difference in the age at diagnosis between our study and two other recently published studies on children with PCD in China [24, 25]. There were no other major differences between Chinese children and adults with PCD in any aspects of clinical presentations or genetics. Our study prompts healthcare providers of adults to consider PCD as a differential diagnosis for patients with related symptoms, especially in basic hospitals where physicians are not well-trained to diagnose rare disorders.

In our study, patients with PCD were confirmed based on Kartagener syndrome (heterotaxis phenotypes), ciliary axonemal defects by TEM, and genetic tests of compound heterozygous or homozygous mutations in known PCD-causing genes. After this confirming procedure, the median age at onset was 1.5 years, which is in accordance with the early onset feature of PCD. Compared to that in a previous study, the prevalence of cough and/or productive cough in this study was similar, but the prevalence of otitis media (13.0%) was significantly less than that in previous studies (weighted mean 74%) [26]. This finding may be because some of our patients forgot their childhood history of otitis media, since they came to PUMCH in their later years. In addition, it is more difficult to suspect PCD in patients who show only otitis media without other obvious respiratory symptoms. The high prevalence of bronchiectasis in our study (95.8%) compared with that in a previous study (weighted mean 56%) suggested that patients with PCD would be more likely to develop bronchiectasis at an older age [26]. Computed tomography showed the predominance of bronchiectasis in the middle and lower lobes, which similarly occurred in a previous study [27].

Our study helps reveal the mutation spectrum and genotype–phenotype relationship in Chinese patients with PCD. Among all candidate PCD-relevant genes, *DNAH5* was the most prevalent disease-causing gene, occurring in 23.1% of all patients with PCD, compared to a previous study by Failly in which only 15% of 89 unrelated individuals of European origin with PCD were associated with *DNAH5* and in a study by Hornef in which 28% of 109 individuals of European or North American origin with PCD carried mutations clustering within exons 34, 50, 63, 76, and 77, and no c.8383C > T variants were observed [20, 28]. In contrast, the c.8383C > T stop-gain mutation in *DNAH5* was a hotspot among patients of Chinese origin with PCD in our study. It is said that *DNAI1*, the initial PCD-implicated gene, occurs in 9% of all patients with identified PCD [6]. To our surprise, no patient had homozygous or compound heterozygous variations in *DNAI1* in our cohort study. *CCDC39* and *CCDC40* are the most prevalent mutated genes in individuals of Egyptian origin with PCD [29]. A comparable phenomenon was observed in 58 Tunisian patients with PCD in whom *CCDC39* and c.2190del in *CCDC39* was a mutation hotspot, whereas deleterious mutations in *CCDC39* or *CCDC40* were rarely observed in Chinese patients with PCD and only one case involved *CCDC40* dysfunction [30]. These results elucidate the disparate genetic spectra of patients of Chinese and other ethnic backgrounds with PCD.

NGS provides us with abundant novel variants without bias as well as a great challenge to determine pathogenic mutations in individuals with PCD. In this cohort, 8 of 32 variations found in our patients were marked as damaging in the HGMD with definite pathogenicity. A total of 24 novel mutations were found, most of which were nonsense, premRNA splicing, and frameshift mutations, and presumably had deleterious effects. The remaining missense variants were either pathogenic/likely pathogenic mutations assessed by ACMG/AMP or VUS with a high damaging score provided by VarCards. As described in the results, VUS, assessed by ACMG standards, could be deemed to be deleterious by VarCards with relatively high damaging scores because of either an extremely low MAF of less than 0.0001 (c.9631C > T (p.P3211S) in *DNAH11*) or location in a relatively conserved domain of protein (c.53T > C (p.I18T) mutation in *LRRC6*), which further provided evidence for their pathogenicity. A more complicated case was patient 23, who carried a c.4596 + 1G > A mutation in *DNAH5* of paternal origin and two missense variants, c.7520G > C (p.W2507S) and c.5518C > T (p.R1840W) of maternal origin. Both missense variations were assumed to be VUS by ACMG but deleterious by VarCards, with c.5518C > T slightly more detrimental (damaging score of 0.61) than c.7520G > C (damaging score of 0.57). However, the possibility of the synergetic effects of both variants cannot be ruled out. Overall, identified variants were classified as disease-causing based on their minor allele frequencies, biological relevance to the disease, bioinformatic tools applied, and previous results published. However, the true effects of those putative deleterious mutations are uncertain, and more functional studies or long-term follow-ups are warranted.

In terms of the relationship between genotypes and axonemal defects in patients with PCD, two cases with mutated *CCNO* had absent or short cilia, consistent with the results of a previous study by Wallmeier that *CCNO* is involved in cilia generation [31]. Patient 15 with pathogenic variations in *DNAH1* had inner dynein defects. This finding can be explained by the *DNAH1* gene function of encoding axonemal inner dynein arms [32]. Dysfunction of *DNAH5* regularly caused outer dynein defects in the ciliary axoneme in a previous report [20]. In our study, however, abnormalities in the axonemal structure were not detected in patient 14, who had deleterious variations in *DNAH5*, perhaps due to inappropriate sampling sites for TEM. In addition, *DNAH11* was thought not to cause any axonemal defects, whereas in patient 25 with compound heterozygous mutations in *DNAH11*, ciliary absence in respiratory epithelial cells was detected, which may also have been due to the sites of sampling and may require repeating TEM examinations of the airway epithelium [33].

## Conclusion

To summarize, we detected pathogenic variants in 26 patients of Chinese origin with PCD by combining WES with low-pass WGS, helping to reveal SNVs, small indels, and large CNVs focusing on selected known and putative PCD-causing genes. This combination was highly efficient and practical, increasing the detection rate to 73.1% in our cohort. A total of 24 novel and 8 HGMD-recorded variants scattered among 8 known PCD-causing genes were identified. *DNAH5* and a nonsense mutation (c.8383C > T, p.R2795X) in this gene showed the highest prevalence in our collection. Despite the small sample size, our study profiled an overview of clinical symptoms and genetic characteristics of Chinese patients with PCD. In addition, this study demonstrated the practical clinical utility of combining WES and low-pass WGS to increase the diagnostic yield for PCD and showed the value of genome and exome sequencing as a diagnostic tool.

## Materials and methods

### Diagnostic criteria for patients with suspected PCD

**Fig. 7:**
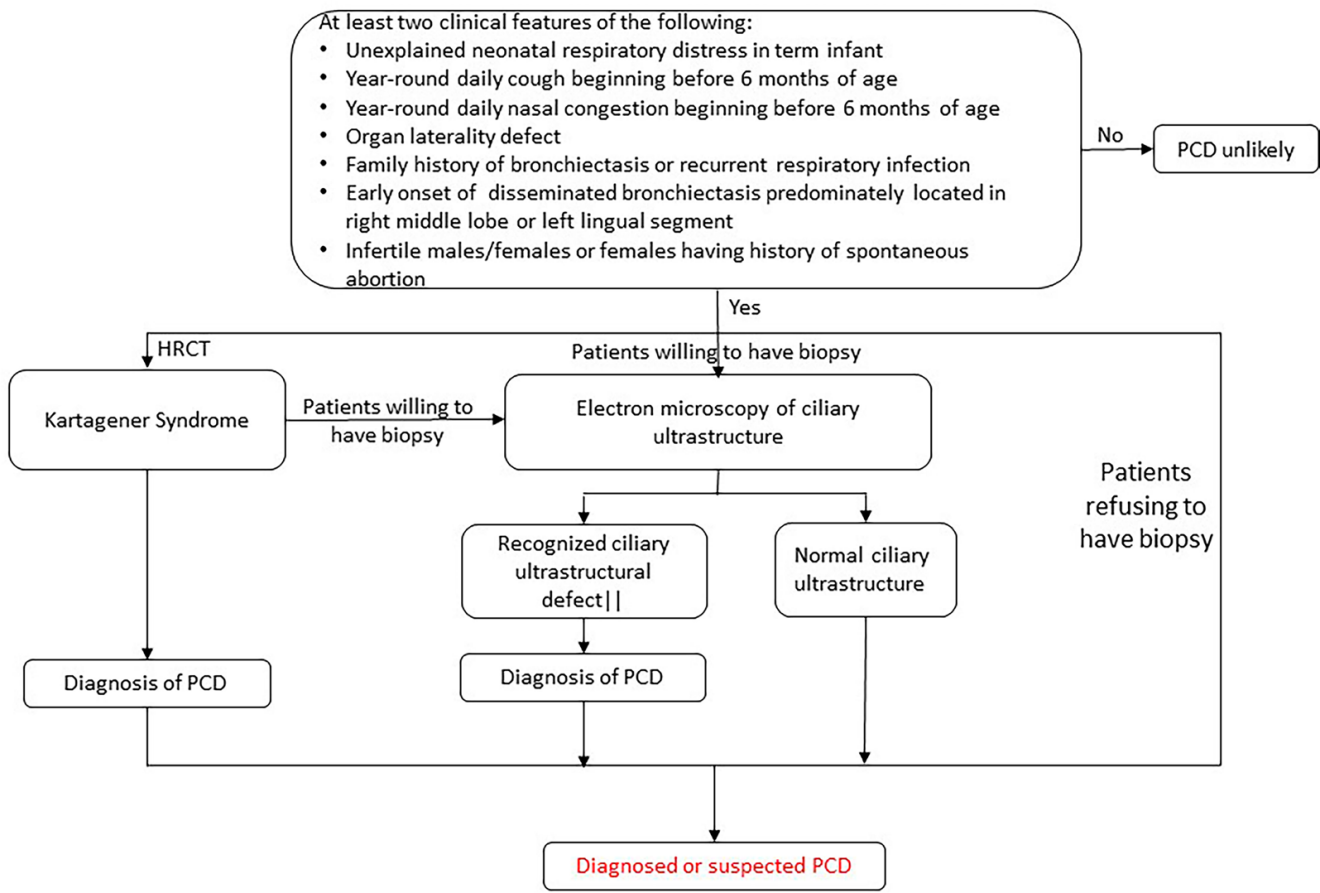
The diagram of PCD diagnostic criteria. Patients with at least two of the following clinical manifestations are highly suspected to have PCD in Peking Union Medical Hospital: (1) Unexplained neonatal respiratory distress in term infant; (2) Year-round daily cough beginning before 6 months of age; (3) Year-round daily nasal congestion beginning before 6 months of age; (4) Organ laterality defect; (5) Family history of bronchiectasis or recurrent respiratory infection; (6) Early onset of disseminated bronchiectasis predominately located in right middle lobe or left lingual segment; (7) Infertile males/females or females having history of spontaneous abortion. High-resolution computed tomography scans (HRCT) were performed for all patients to evaluate bronchiectasis and KS. For those patients who were willing to have biopsy, TEM for respiratory epithelial cilia was performed for PCD diagnosis. For patients who decline endobronchial biopsy, WES and/or low-pass WGS are convenient and reliable ways to diagnose PCD

We performed a retrospective review of patients who attended Peking Union Medical Hospital between 2012 and 2020, who were highly suspected of having PCD on the basis of the following clinical manifestations: (1) unexplained neonatal respiratory distress in term infant; (2) year-round daily cough beginning before 6 months of age; (3) year-round daily nasal congestion beginning before 6 months of age; (4) organ laterality defect; (5) family history of bronchiectasis or recurrent respiratory infection; (6) early onset of disseminated bronchiectasis predominately located in right middle lobe or left lingual segment; (7) infertile males/females or females having history of spontaneous abortion (as narrated in Fig. 7). Pulmonary function tests, high-resolution computed tomography scans (HRCT) for the chest and nasosinus, TEM for respiratory epithelial cilia, sperm tests (adult male patients only) and WES and/or low-pass WGS were performed for diagnosis and evaluation. Definitive PCD diagnosis was established when the patient met at least one of the following conditions: (1) biallelic putative pathogenic mutations in known PCD-causing genes, (2) typical ciliary ultrastructural defects on transmission electron microscopy, or (3) PCD-related clinical manifestations with a family history of PCD. Patients who had Kartagener syndrome and related clinical symptoms were considered to have PCD even if the abovementioned criteria were not completely fulfilled [25]. Patients who had been diagnosed with KS did not undergo TEM for cilia if they were reluctant to do so. In our cohort, 26 patients of Chinese origin with confirmed PCD underwent peripheral blood sample collection. More clinical details were documented in Additional file 2: Table S1. Informed consent was acquired from the patients, and all methods were approved by the institutional review board committee at Peking Union Medical College.

### Whole-exome sequencing (WES) and putative SNV detection

Genomic DNA was extracted from the blood samples of the 26 patients with PCD and their parents, if available, using a QIAamp DNA Blood Mini Kit (QIAGEN, Hilden, Germany), followed by whole-exome sequencing for an average sequencing depth of 100× (100× WES). WES was conducted on the Illumina HiSeq XTen platform, and the reads were aligned to Genome Reference Consortium Human Build 37 (GRCh37/hg19) human assembly using the Burrows-Wheeler Aligner (bwa) [34]. SNVs, small insertions and deletions (indels) were analyzed using SAMtools and ANNOVAR software [35, 36]. SIFT, Polyphen2, MutationTaster, and CADD were chosen to predict variant pathogenicity [37–40]. The OMIM, Clinvar, HGMD, KEGG, PID, and REACTOME_PATHWAY databases were searched for functional annotation [41–46]. Sanger sequencing was performed to avoid false positive outcomes of WES and to verify the parental origins of the mutations. In out cohort, the price of WES and low-pass WGS was 1500RMB and 2100RMB, respectively, for each patient. All costs arising from WES and WGS were paid by our institution, with no costs incurred by the patients themselves.

### Variant-level inclusion and exclusion criteria

To prioritize high pathogenic variants underlying a rare autosomal recessive disease, all detected SNVs and small indels with a minor allele frequency (MAF) of greater than 0.01 in any database of the 1000 genomes Project (https://www.1000genomes.org), NHLBI GO Exome Sequencing Project Exome Variant Server (ESP6500SI-V2) (http://evs.gs.washington.edu/EVS), and GnomAD (https://gnomad.broadinstitute.org/) were filtered out [47, 48]. Variants in the exonic region and splicing region ± 10 bp of the adjacent exon were further analyzed. Synonymous variants were excluded. Loss-of-function variants, such as nonsense, frameshift and splicing-effecting mutations were prioritized, as they are more likely to damage transcribed and translated products. For in silico prediction, all point mutations in the exonic region, apart from those adjacent to the splicing region that may affect premRNA splicing, were predicted to be pathogenic/likely pathogenic, benign/likely benign, or of uncertain significance based on the ACMG/AMP 2015 guideline criteria using InterVar (http://wintervar.wglab.org/) and VarCards (http://159.226.67.237/sun/varcards/welcome) [49, 50]. For small indels in the exonic region, MutationTaster (http://www.mutationtaster.org/) was chosen to annotate their effects [39]. The possible damaging effect of all variants in the splicing junction 10 bp was predicted by Human Splicing Finder (http://www.umd.be/HSF3/index.html). The Human Gene Mutation Database (HGMD) was accessed to distinguish between known PCD-relevant variants and potentially pathogenic novel variants [43].

### Candidate gene selection criteria

**Fig. 8:**
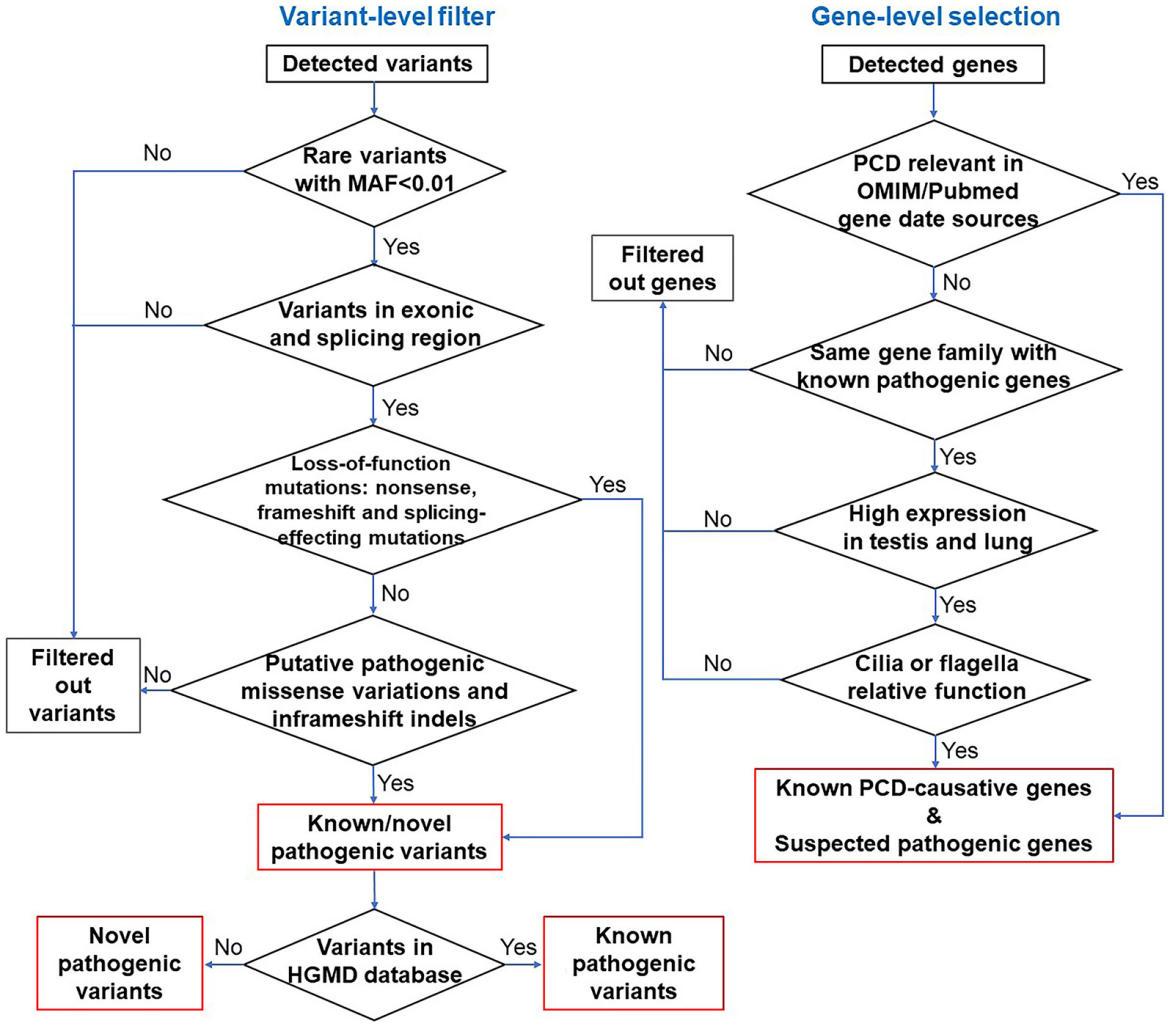
The variant-level inclusion and exclusion diagram and candidate pathogenic gene selection diagram. For functional effect assessment, synonymous variants were filtered out. Loss-of-function variants, such as nonsense, frameshift and splicing-effecting mutations were prioritized. Putative pathogenic missense variations and frameshift indels were kept

A total of 51 known PCD-causative genes were retrieved from OMIM and PubMed (listed in the supplemental file, Additional file 1). Other potentially pathogenic genes were selected based on the following features: (1) belonging to the same gene family as known PCD pathogenic genes; (2) high expression in lung and testis tissues, where motile cilia are abundant, either for mucus expelling or for sperm motility; and (3) encoding a ciliary structural protein or the functional composition of motile cilia, such as proteins associated with intraflagellar transport (IFT) or cilia stability and assembly [5]. With the criteria, 42 suspected PCD genes were selected in our study as the first group of candidates of focus. Next, either compound heterozygous or homozygous variants in these 93 candidate genes were selected as the second group of candidates for further assessment. (The diagram of candidate variants and gene selection is shown in Fig. 8).

### Low-pass WGS and putative CNV detection

**Fig. 9:**
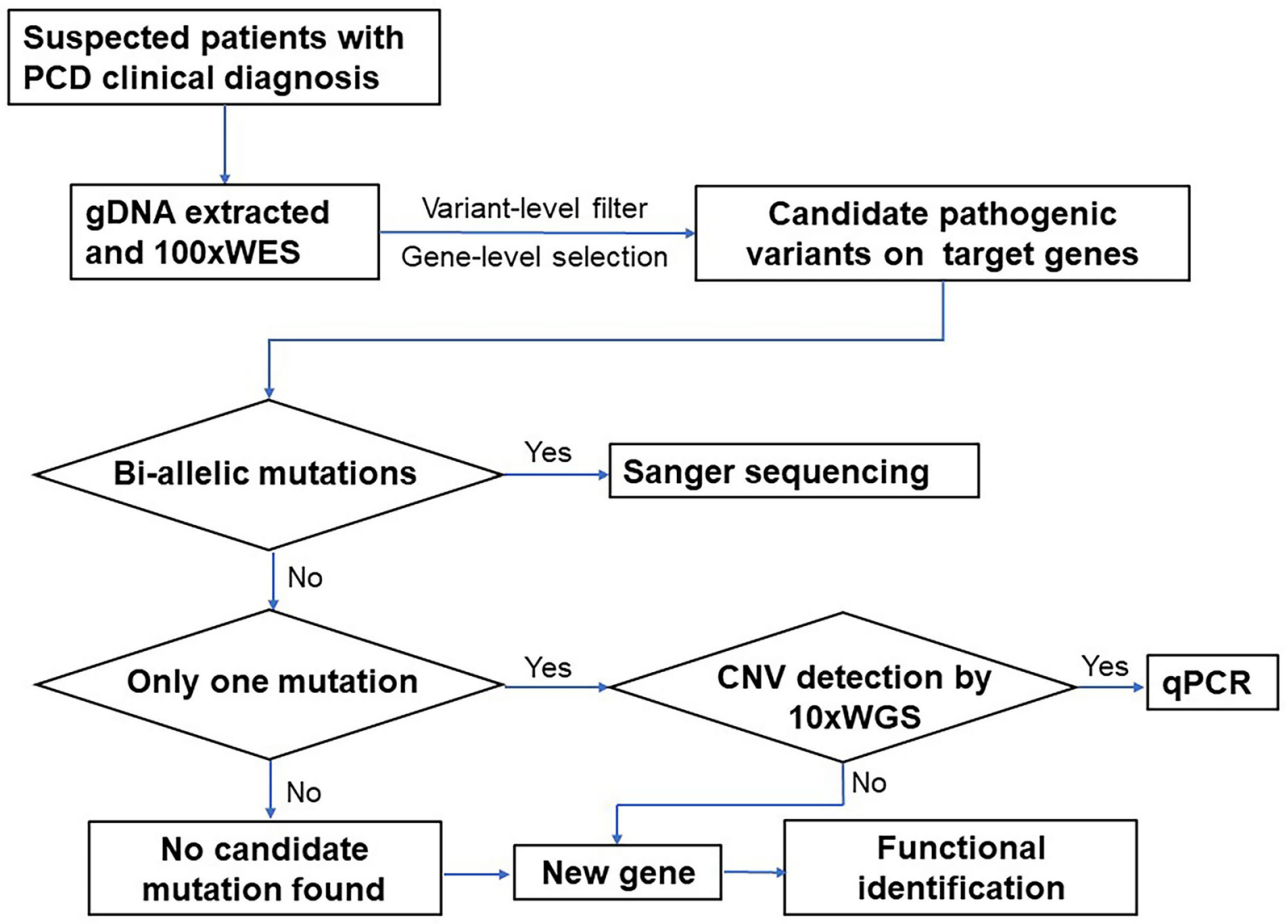
The diagram of variant detection in suspected PCD patients by WES and/or low-pass WGS in combination. Detected biallelic mutations in candidate genes, which should be either homozygous or compound heterozygous, were verified by Sanger sequencing. If only one variant in a certain candidate gene was found that was confirmed to be pathogenic by ACMG, Low-pass WGS was performed for targeted CNV analysis to detect the second hit. These patients and their parents, if available, also underwent qPCR to ascertain detected CNVs. For those unsolved cases, new genes may be implicated, and further analysis and functional identification are necessary

As WES is not sensitive to large DNA segmental duplications and deletions, WGS with an average sequencing depth of 10× (10× WGS) was performed when only one highly suspected pathogenic variant was detected in 1 of the 93 candidate genes listed above [51]. CNVs were analyzed using CoNIFER (V0.2.2) [52]. StringentLib, InclusiveLib, CNVD, and Database of Genomic Variants (DGV) Gold Standard Variants (July.2015) were chosen for CNV annotation. Quantitative polymerase chain reaction (qPCR) on the gDNA of patients and available parents was conducted using Hieff qPCR SYBR Green Master Mix (YEASEN) to verify CNVs and their parental origin (Fig 9).

## Supplementary Information

**Additional file 1**. The gene list of known 51 PCD genes and 42 suspected PCD genes. 93 PCD candidate genes are listed in the Excel file, including 51 known genes and 42 suspected ones. Fifty-one known PCD-causative genes were retrieved from OMIM and PubMed, and forty-two suspected genes were selected based on all of the following features: (1) belong to the same gene family as known PCD pathogenic genes; (2) have high expression in lung and testis tissues, where motile cilia are abundant, either for mucus expelling or for sperm motility; (3) encode a ciliary structural protein or a functional composition of motile cilia, such as proteins associating with intraflagellar transport (IFT) or cilia stability and assembly.

**Additional file 2**. Table S1. Clinical features and detected mutated genes and variants of the 26 confirmed PCD patients of Chinese origin. a. The patients are listed according to different categories outlining the included patients. The first thirteen patients (patient 1, 2, 3, 4, 5, 6, 13, 18, 20, 22, 24, 25, 26) have KS (with or without ciliary ultrastructural defects), the next six patients (patient 8, 9, 10, 11, 12, 15) have ciliary ultrastructural defects without KS, and the last seven patients (patient 7, 14, 16, 17, 19, 21, 23) are patients with biallelic mutations alone (without KS or any ciliary ultrastructural defect). b. Most of the patients are too young that we did not know whether they have the infertility history or let them do the sperm test. c. If family members of a patient have a history of asthma or chronic bronchitis, the patient is determined to have a suspected PCD family history. d. For bronchiectasis, 1 means the patient has bronchiectasis in that certain location shown in CT scans, while 0 means no bronchiectasis is observed in that certain location. e. For pathogenicity prediction, SNVs in exonic regions are predicted by ACMG/AMP, indels are predicted using MutationTaster, variants in splicing regions are predicted using Human Splicing Finder. f. NA: not available, ND: not detected

## Data Availability

The datasets supporting the conclusions of this article are included within the article and its additional files.

## Abbreviations

CNV: Copy number variant
DLCO: Diffusing capacity of the lungs for carbon monoxide
DGV: Database of genomic variants
FEV_1_: Forced expiratory volume in 1 s
FVC: Forced vital capacity
HGMD: Human gene mutation database
HRCT: High-resolution computed tomography scan
HSVA: High-speed video microscopy with ciliary beat pattern analysis
IF: Immunofluorescence
IFT: Intraflagellar transport
KS: Kartagener syndrome
MAF: Minor allele frequency
nNO: Nasal nitric oxide
PCD: Primary ciliary dyskinesia
PUMCH: Peking Union Medical College Hospital
qPCR: Quantitative polymerase chain reaction
SNV: Single nucleotide variation
TEM: Transmission electron microscopy
TLC: Total lung capacity
VUS: Variants of uncertain significance
WES: Whole-exome sequencing
WGS: Whole-genome sequencing
